# Supplementary material for: Acute kidney injury in COVID-19 pediatric patients in North America: Analysis of the virtual pediatric systems data
Source: PLoS One. 2022 Apr 26;17(4):e0266737. doi: 10.1371/journal.pone.0266737 (PMC9041802; doi:10.1371/journal.pone.0266737)
Supplement: S1 File — (DOCX) [file pone.0266737.s001.docx]

**Supporting Information**

**S1 Table A:** Epidemiology of COVID-19 Pediatric Patients**.**

| **Study** | **Location** | **Total COVID Cases** | **Mean Age (years, IQR)** | **AKI Incidence (n, %)** | **AKI Patients Admitted to the ICU** | **AKI Patient’s Use of Renal Support** | **Mortality**  **(n, %) COVID +** | **Mortality**  **(n, %) COVID + & AKI +** |
| --- | --- | --- | --- | --- | --- | --- | --- | --- |
| VPS | North America | 2,597 | N/A | 297 (10.7%) | 297 (100%) | 15 (5.4%) | 58 (2.2%) | 21 (7.5%) |
| CDC | United States | 149,760 | N/A | 2,572 (1.7%) | 15 (0.58%) | N/A | N/A | N/A |
| Godfred-Cato S et al. [19] | United States | 570 | 8 (4-12) | 105 (18%) | N/A | 2 (0.4%) | 10 (1.8%) | N/A |
| Dionne et al. [20] | United States | 25 | 9.7 (2.7-15.0) | 2 (8%) | 2 (100%) | N/A | 0 (0%) | 0 (0%) |
| Derespina et al. [21] | United States | 70 | 15 (9-19) | 9 (12.9%) | 9 (100%) | 1 (1.4%) | 2 (2.8%) | N/A |
| Livingston & Bucher [8] | Italy | 22,512 | N/A | 270 (1.2%) | N/A | N/A | 1625 (7.2%) | N/A |
| Tagarro et al. [9] | Spain (Madrid) | 4,695 | 3 (.9-6) | 41 (0.8%) | 4 (9.7%) | N/A | 0 (0%) | 0 (0%) |
| Toubiana et al. [14] | France | 21 | 7.9 | 11 (52%) | N/A | N/A | 0 (0%) | 0 (0%) |
| Grimaud et al. [15] | France | 20 | 10 | 14 (70%) | 14 (100%) | 0 (0%) | 0 (0%) | 0 (0%) |
| Whittaker et al. [17] | England | 58 | 9 (5.7-14) | 13 (22%) | 13 (100%) | N/A | 1 (2%) | N/A |
| Stewart et al. [27] | England | 52 | 9.4 (5.6-12.9) | 15 (29%) | 14 (93%) | 0 (0%) | 0 (0%) | 0 (0%) |
| Bjornstad et al. [12] | United States, Western Europe, Eastern Europe/Russia | 106 | 11 | 47 (44%) | N/A | 0 (0%) | 3 (5%) | 3 (6%) |
| Dong et al. [11] | China | 80,174 | 7 (2-13) | 2,143 (2.7%) | 13 (0.6%) | N/A | 1 (.001%) | N/A |
| Wang et al. [22] | China | 238 | N/A | 3 (1.3%) | 3 (100%) | 3 (100%) | N/A | 1 (33%) |
| Mamishi et al. [16] | Iran | 45 | 7 (4-9.9) | 13 (29%) | N/A | 0 (0%) | 5 (11%) | N/A |
| Kari et al. [13] | Saudi Arabia | 89 | AKI group: 4.5  Non-AKI group: 6.2 | 19 (21%) | 6 (32%) | 0 (0%) | 0 (0%) | 37 (42%) |

**S1 Table B** Variable Definitions. Definitions were adapted from https://covid19.myvps.org/

| **Variable** | **Definitions** |
| --- | --- |
| COVID-19 positive | Patients with a positive COVID-test that were treated in a North American PICU (Earliest PICU care day following COVID+ test result). Existing PICU patients were identified on the day of the COVID+ test result. Patients that were previously at home or in a non-PICU setting were identified on the day of PICU admission. |
| Confirmed Deaths | Patients with a positive COVID-19 test that had been treated in a North American PICU and who died during their admission. |
| PICU Days | Cumulative burden of COVID+ patients on PICU bed utilization over time. This was defined as receiving care in a PICU and having a COVID+ test result. A single patient could contribute multiple values to this metric, equal to the number of days they have received care in a PICU. |
| Therapies Used | The cumulative burden on respiratory therapy resources used in the PICU over time. This is the maximum level of respiratory support for each patient, for each PICU day. A single patient could contribute to multiple values to this metric, equal to the number of days they have received care in a PICU> |
| Comorbidity of Patients | The severity of comorbidities (if any) of patients with positive COVID-19 tests that have been treated in North American PICUs. Severity was defined as either “none,” “mild,” or “moderate/severe,” based upon the clinical assessment of the submitting site. |
| Cumulative COVID-19 Positive PICU Admission | The total to date of all COVID-19 positive patients admission to the PICU. The cumulative total only considered admissions. Deaths or discharges did not impact the metric. |
| COVID-19 Positive PICU Admissions per Day | The total unique patient admissions to PICUs on each day. |
| Average of LOS by Age Group | The average length of stay in the PICU in days of each age group. |
| Average of LOS by Prior Comorbidities | Identifies the average length of stay in the PICU in days based on the severity of comorbidities (none, mild, or moderate/severe) the patient had. |
| Organ Support | Identifies the organ support provided for each patient. A patient may have had multiple organ therapies. For instance, if a patient received both vasoactive infusions and steroids, the patient would be counted once in the vasoactive infusions and once in the steroid category. Not all patients have this data as this question was added after submissions started. |

**S1 Table C: Adjusted association of different variables on the outcomes**

| **Continuous outcome** | | | | | | | | |
| --- | --- | --- | --- | --- | --- | --- | --- | --- |
| Variables | | Un-standardized Coefficients (95% CI) | | | | | | |
|  |  | Hospital LOS (days) | | PIM 2 Probability of Death (%) | | | PIM 3 Probability of Death (%) | |
| Age (continuous) | | 0.04 (-0.54 - 0.62) | | -0.18 (-0.41 - 0.04) | | | -0.17 (-0.4 - 0.05) | |
| Cardiovascular comorbidity (yes) | | 3.47 (1.93 - 5.01)* | | 1.97 (1.37 - 2.57)* | | | 1.51 (0.92 - 2.11) | |
| Endocrine comorbidity (yes) | | 2.63 (0.76 - 4.49) | | 1.12 (0.39 - 1.84) | | | 0.88 (0.16 - 1.6) | |
| Hematology comorbidity (yes) | | 3.72 (2.03 - 5.42)* | | 0.99 (0.33 - 1.65) | | | 1.25 (0.6 - 1.9) | |
| Respiratory comorbidity (yes) | | 4.91 (3.46 - 6.36)* | | 1.61 (1.04 - 2.17)* | | | 1.38 (0.82 - 1.93) | |
| AKI (yes) | | 6.29 (3.95 - 8.64)* | | 0.81 (-0.11 - 1.72) | | | 0.61 (-0.3 - 1.51) | |
| **Categorical outcome** | | | | | | | | |
| Variables | Adjusted odds ratio (95% CI) | | | | | | | |
|  | Mortality | | Airway / Respiratory support | | Cardio-respiratory support | Kidney support | | Vascular access |
| Age (continuous) | 0.92  (0.73 - 1.16) | | 0.94  (0.86 - 1.02) | | 0.83  (0.61 - 1.13) | 1.86  (1.1 - 3.13)* | | 1.13  (1.05 - 1.22)* |
| Cardiovascular comorbidity (yes) | 2.44  (1.34 - 4.45)* | | 1.19  (0.97 - 1.48) | | 35.22  (4.7 - 263.78)* | 23.12  (3 - 178.1)* | | 2.41  (2.01 - 2.91)* |
| Endocrine comorbidity (yes) | 3.85  (2.17 - 6.84)* | | 0.81  (0.62 - 1.06) | | 0.56  (0.16 - 1.93) | 0.48  (0.13 - 1.73) | | 0.84  (0.66 - 1.08) |
| Hematology comorbidity (yes) | 2.12  (1.21 - 3.72)* | | 0.82  (0.66 - 1.04) | | 1.44  (0.65 - 3.2) | 2.01  (0.8 - 5.09) | | 2.45  (2 - 2.99)* |
| Respiratory comorbidity (yes) | 11.63  (3.59 - 37.75)* | | 21.24  (16.91 - 26.69)* | | 14.93  (2.01 - 111.05)* | 1.11  (0.41 - 2.97) | | 1.7  (1.41 - 2.05)* |
| AKI (yes) | 2.69  (1.48 - 4.88)* | | 1.61  (1.16 - 2.24)* | | 1.88  (0.77 - 4.56) | 5.34  (2.15 - 13.25)* | | 3.51  (2.63 - 4.7)* |

*p<0.05

AKI: Acute Kidney Injury; LOS: Length of stay; PIM: Pediatric index of mortality; IQR: Interquartile range; CI: Confidence Interval

Adjusted for variables such as age (continuous in years), presence of cardiovascular, endocrine, hematology, and respiratory co-morbidities (categorical) and AKI (categorical).

**S1 Figure A:** Association of different categorical outcomes among AKI patients across different AKI stage

AKI: Acute Kidney Injury

**S1 Figure B :** Association of different continuous outcomes among AKI patients across different AKI stage

AKI: Acute Kidney Injury; LOS: Length of stay; PIM: Pediatric index of mortality
