# Supplementary material for: Acute kidney injury in COVID-19 pediatric patients in North America: Analysis of the virtual pediatric systems data
Source: PLoS One. 2022 Apr 26;17(4):e0266737. doi: 10.1371/journal.pone.0266737 (PMC9041802; doi:10.1371/journal.pone.0266737)
Supplement: Attachment — Submitted filename: Response to Reviewers.docx [file pone.0266737.s003.docx]

**Journal Requirements:**

1. Please ensure that your manuscript meets PLOS ONE's style requirements, including those for file naming.

**Response: The manuscript has been reviewed and the appropriate changes in accordance with PLOS ONE’s style requirements have been made.**

2. Please review your reference list to ensure that it is complete and correct. If you have cited papers that have been retracted, please include the rationale for doing so in the manuscript text, or remove these references and replace them with relevant current references. Any changes to the reference list should be mentioned in the rebuttal letter that accompanies your revised manuscript. If you need to cite a retracted article, indicate the article’s retracted status in the References list and also include a citation and full reference for the retraction notice.

**Response: The reference list has been reviewed.**

3. In your Data Availability statement, you have not specified where the minimal data set underlying the results described in your manuscript can be found. PLOS defines a study's minimal data set as the underlying data used to reach the conclusions drawn in the manuscript and any additional data required to replicate the reported study findings in their entirety. All PLOS journals require that the minimal data set be made fully available.

Upon re-submitting your revised manuscript, please upload your study’s minimal underlying data set as either Supporting Information files or to a stable, public repository and include the relevant URLs, DOIs, or accession numbers within your revised cover letter.

Any potentially identifying patient information must be fully anonymized.

Important: If there are ethical or legal restrictions to sharing your data publicly, please explain these restrictions in detail.

**Response: The authors have uploaded the minimal data set underlying the results described in the manuscript as a Supporting Information file.**

4. LOS requires an ORCID iD for the corresponding author in Editorial Manager on papers submitted after December 6th, 2016. Please ensure that you have an ORCID iD and that it is validated in Editorial Manager.

**Response: The ORCID iD for the corresponding author has been updated.**

5. Please include your full ethics statement in the ‘Methods’ section of your manuscript file. In your statement, please include the full name of the IRB or ethics committee who approved or waived your study, as well as whether or not you obtained informed written or verbal consent. If consent was waived for your study, please include this information in your statement as well.

**Response: The authors’ ethics statement has been included in the manuscript file. Kindly view this statement in lines 109 – 112.**

**Review’s Comments:**

**Reviewer 1**

**Reviewer Comment**: It is usually best to leave continuous variables uncategorized, except where such categorization are standard e.g categorizing blood sugar levels according to diabetes defining cut-off values. It seems arbitrary that in some of the analyses, age is categorized as <12 years or >12 years (line 116) while in other instances it is categorized as <2 years or >2 years (line 146 and Table 1). The <2 years or >2 years categorization lumps up individuals with different anatomic/developmental and physiologic profiles. The group >2 years would comprise for instance 3-5 year olds, 6-10 year olds, young adolescents and late adolescents, given the cut off age in the study of <21 years. If the age is left as a continuous variable, you could then assess what the odds ratios would be for different ages with incremental age and this would provide more clinically applicable and relevant deductions. Kindly have a look at this.

**Response: We thank the reviewer very much for their suggestion. We have re-done our data analysis as suggested so that age may be analyzed as a continuous variable with odds ratios. Kindly view the changes in the statistical analysis portion of the methods section, the results section, Table 1, and Table 3 of the manuscript. We have included S3 Table under supporting information entitled “Adjusted association of different variables on the outcomes” so that the adjusted association of age as a continuous variable with the different outcomes is shown.**

**Reviewer Comment**: In line 168, you mention "circulatory dysfunction" and "vascular dysfunction". In reading through the manuscript I did not come across where these entities had been clearly defined and distinguished.

**Response: We thank the reviewer for pointing out this issue.** **By using the terms ‘circulatory’ and ‘vascular’ dysfunction, we are using the terminology set forth by the Virtual Pediatric Systems (VPS) database and do not have any further information on how these terms were distinguished. According to the VPS data set, the organ systems involved by COVID-19 identifies organ specific symptoms a patient in the database was reported to have.**

**Reviewer 2**

**Comment:** Thank you for your submission. I am curious to know why pediatric age rage was unto 21-years? It may be important to know that the studies that you have refereed/compared to have used the similar upper age. In line 15 PIM abbreviation is used for the first time without its full name kindly correct.

**Response:** **We thank the reviewer for their comment. According to a 2017 policy statement from the American Academy of Pediatrics (AAP) entitled “Age Limit of Pediatrics,” by Amy Hardin and Jesse Hackell, the AAP acknowledges the upper age limit of pediatrics to be 21 years of age. In accordance to this statement, the US Department of Health and the Food and Drug Administration (FDA) reference approximate age ranges for late adolescence to be 18-21 years old. One very similar study we have referenced and cited as reference 38 in our manuscript entitled “Acute Kidney Injury in Critically Ill children and young adults with suspected SARS-CoV2 infection” by authors Basu et al., utilized an even broader age range of >1 week and <25 years of age. Ultimately, we chose the age of 21 due to the AAP, US Department of Health, and FDA guidelines.**

**Additionally, we thank the reviewer for pointing out the oversight in line 15. The abbreviation has been defined in the manuscript.**
